# Reconstruction of defects of maxillary sinus wall after removal of a huge odontogenic lesion using prebended 3D titanium-mesh and CAD/CAM technique

**DOI:** 10.1186/1746-160X-7-21

**Published:** 2011-11-09

**Authors:** Marcus Stoetzer, Majeed Rana, Constantin von See, André M Eckardt, Nils-Claudius Gellrich

**Affiliations:** 1 Department of Craniomaxillofacial Surgery, Hannover Medical School, Hannover, Germany

**Keywords:** Computer-assisted surgery, rapid prototyping, ondontogenic lesion

## Abstract

A 63 year-old male with a huge odontogenic lesion of sinus maxillaris was treated with computer-assisted surgery. After resection of the odontogenic lesion, the sinus wall was reconstructed with a prebended 3D titanium-mesh using CAD/CAM technique. This work provides a new treatment device for maxillary reconstruction via rapid prototyping procedures.

## Background

Loss of hard and soft tissue structures of the midface due to resection of odontogenic lesions can be associated with substancial functional and aesthetic deficits [1]. Conservative treatment due to simple resection and primary soft-tissue clouser will result in loss of soft and hard tissue support. Radical excision of tumor followed by adequate reconstruction can improve survival and provide more satisfactory functional and aesthetical outcome. Reconstruction of large maxillary defects following ablative surgery could be done by using vascularized bone transfer or, more often, primarily with simultaneous or delayed bone grafting [2]. Another option for maxillary reconstruction might be the use of computer-assisted prebended titanium meshes, which acts as physical three dimensional supports of soft and hard tissue [3]. Computer aided design/modeling (CAD/CAM) software that allows "mirroring" planning coupled to navigation systems has dramatically improved surgical strategies in reconstructive surgery of the craniomaxillofacial skeleton [4]. A major challenge in planning procedures is that virtual reconstruction is based on a true-to-original reconstruction with a patient specific implant. In fact, application of commercially available 3D planning software in the clinical routine suffers from poor handling and an insufficient workflow. Since these systems do not represent a complete solution, they have to be supplemented by additional software and hardware devices.

The purpose of this technical note is to present a novel solution for computer-assisted planning of reconstruction of maxillary defects which is based on combination of user-friendly 3D planning software, iPlan 3.0, (Brainlab^®^, Feldkirchen, Germany) and a consistent physical design of predefined titanium mesh via rapid prototype modeling and CAD/CAM technique [5,6].

## Materials and methods

**Figure 1:**
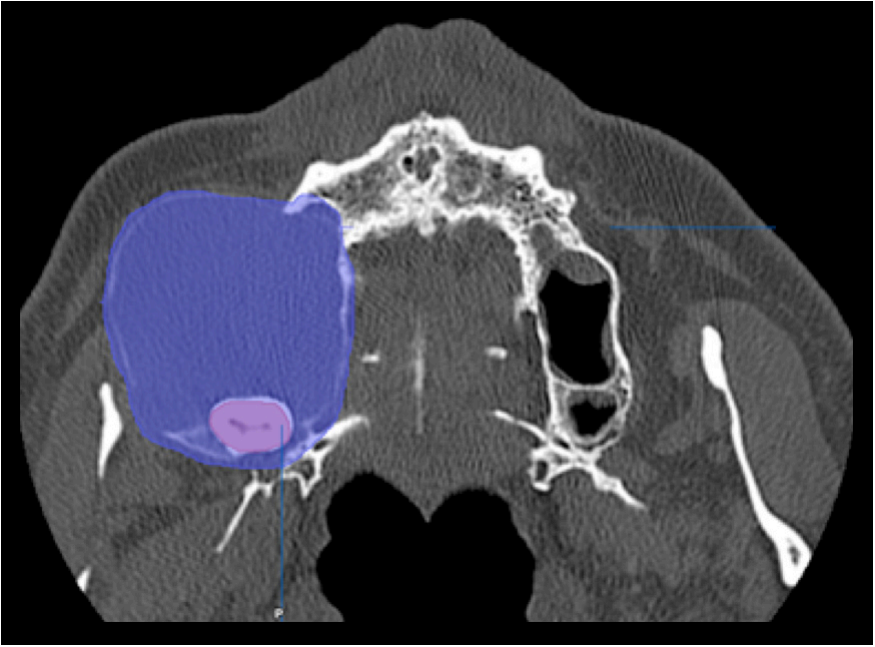
**Axial view of the odontogenic tumor with an extensive bone loss of maxillary alveolar crest and extension to maxillary sinus and nasal cavity (blue)**.

**Figure 2:**
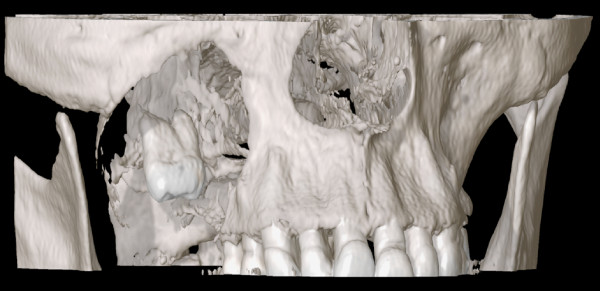
**Display of the computer tomograpy data set in 3D view**. Demonstrating a bone defect caused by the right third molar.

**Figure 3:**
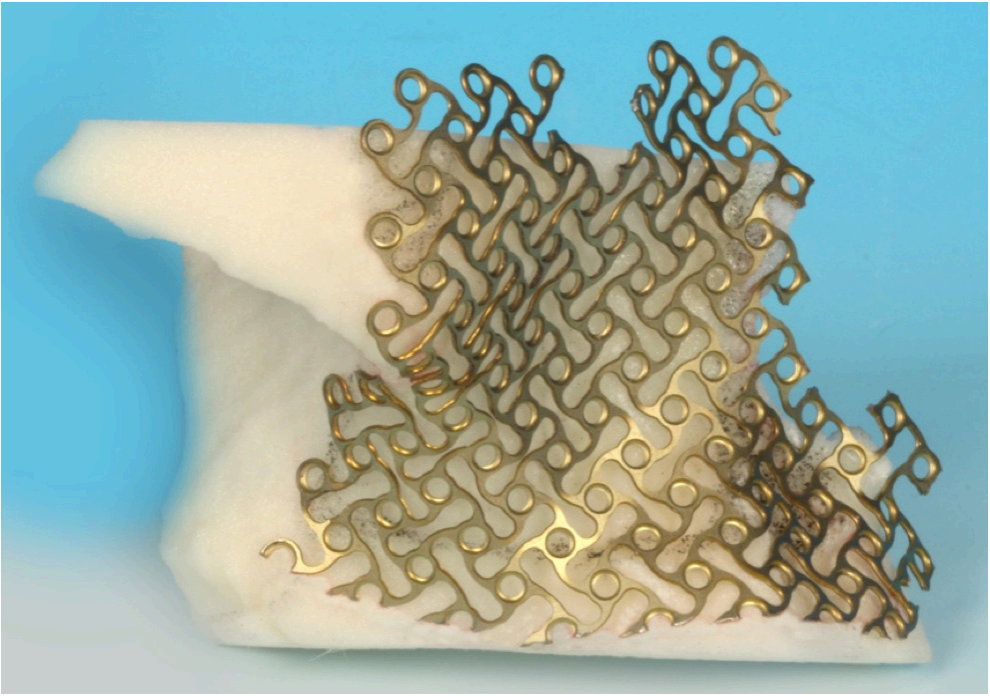
**A stereolithographic (STL) midfacial skull model, produced via rapid prototype modelling**. Prebended titanium mesh (Synthes^®^) for reconstruction of the maxillary sinus wall.

To demonstrate this novel approach, we show an example of surgical planning and treatment of a 63 year old male with a huge slowly growing follicular cyst caused by the right third molar with no clinical symptoms, extensive bone loss of maxillary alveolar crest and extension to maxillary sinus and nasal cavity (Figure 1). Cone beam computer tomography data were transferred into the application software, iPlan 3.0, and reconstructed into 3D images (Figure 2). Accordingly the automatic atlas-based algorithm was used to virtually design anatomical region of zygoma and the maxilla for surgical reconstruction. Virtual planning was performed by using mirrored template of the non-affected left side. Outcome of the virtual planning was a virtual template of the maxilla (STL-file format) for production of a corporeal stereolithographic model. After prototyping of the model (Phacon, Leipzig, Germany) a conventional 3D titanium mesh (Synthes^®^, Umkirch, Germany) was bent to bridge the prospective maxillary defect (Figure 3).

## Results and Discussion

**Figure 4:**
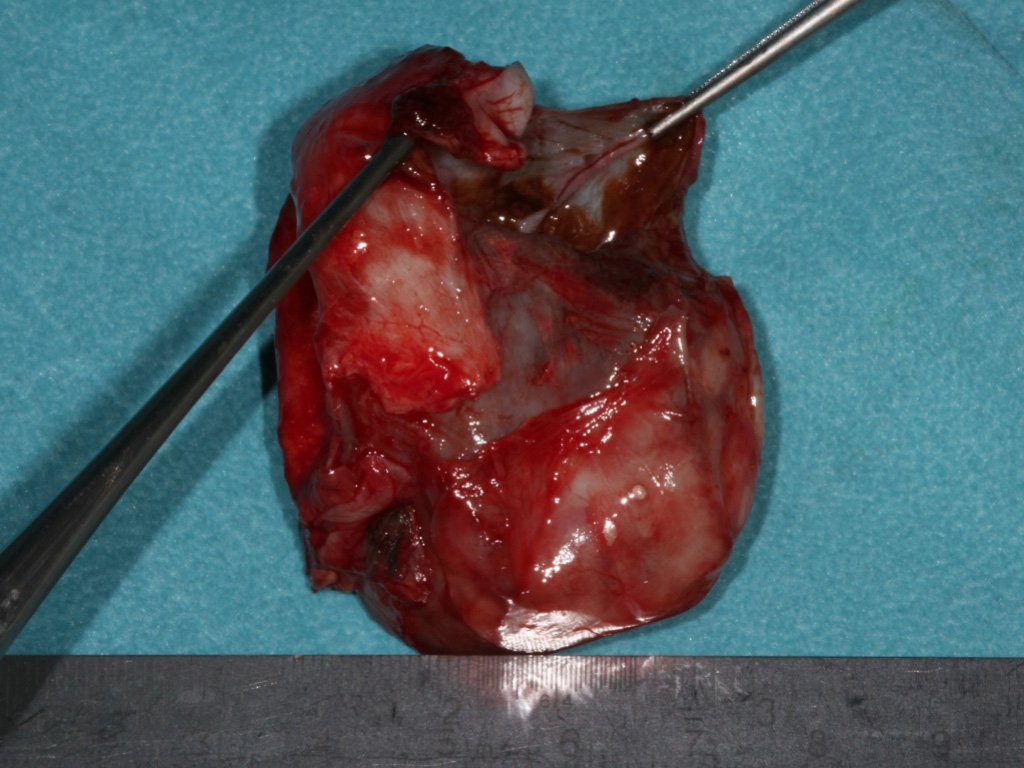
**Excavated five cm huge follicular odontogenic cyst**.

Following removal of the cyst the preformed 3D titanium mesh was used for the osteosynthesis and defect. The pathology report confirmed the diagnosis of a follicular odontogenic cyst (Figure 4).

**Figure 5:**
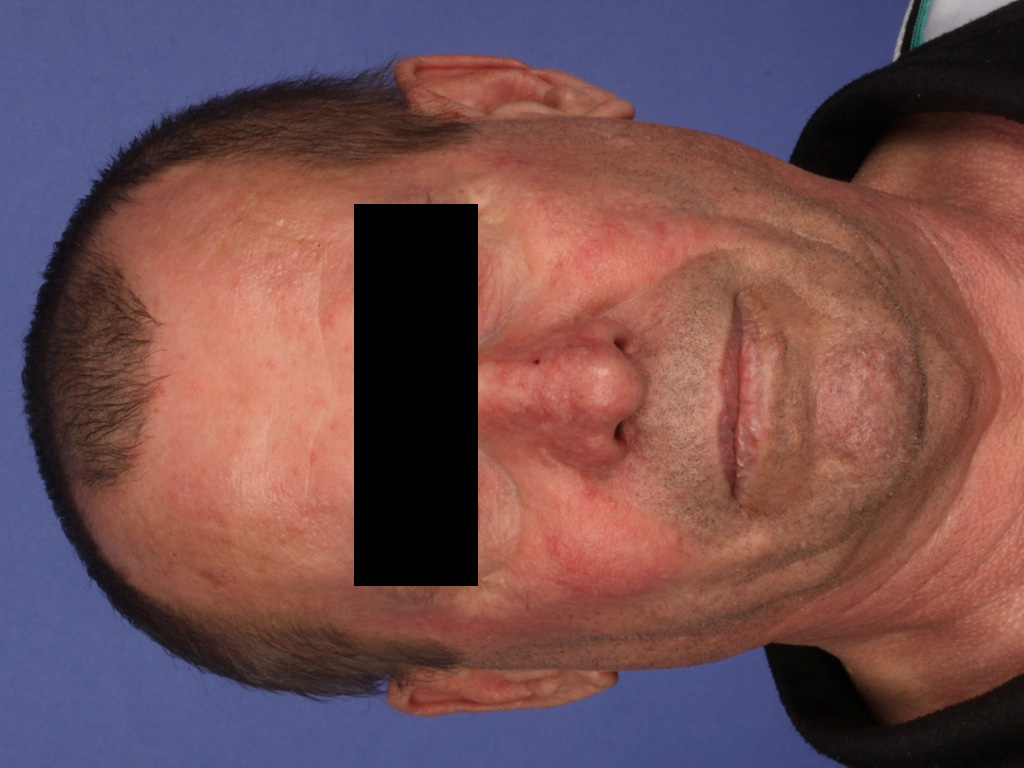
**Postoperative front view of a 63 year old male after reconstruction**.

For quality control the position of the titanium mesh was intraoperatively guided by a navigation system. Finally the accuracy of the virtual simulation was evaluated by a direct comparison of the 3D prediction with the postoperative result. The operation went without complications and secondary reconstruction could be planned. Titanium mesh profile created good visual appearance of the midface (Figure 5).

Many studies in oral and maxillofacial surgery have addressed the benefit of computer-assisted surgery in treatment of head and neck tumors [7,8]. Image-guided tumor resection and reconstruction is a well-established procedure at our institution for complex craniofacial procedures. Based on our case, we could demonstrate that computer-assisted surgery is an eligible and precise method for treatment of huge odontogenic lesions of sinus maxillaris. Regarding the advantages of computer-assisted surgery, this technique will play a major part in craniofacial reconstructive surgery.

## Conflict of interests statement

The authors declare that they have no competing interests.

## Authors' contributions

MS, MR, CS, AME and NCG conceived of the study and participated in its design and coordination. MS and MR made substantial contributions to data acquisation and conception of manuscript. MS and MR drafted and designed the manuscript and contributed equally to this work. NCG and MRU were involved in revising the manuscript. All authors read and approved the final manuscript.

## Consent statement

Written informed consent was obtained from the patient for publication of this case report and accompanying images. A copy of the written consent is available for review by the Editor-in-Chief of this journal.

## Funding

The article processing charges are funded by the Deutsche Forschungsgemeinschaft (DFG), "Open Acess Publizieren".
